# 16S rRNA gene metabarcoding and TEM reveals different ecological strategies within the genus *Neogloboquadrina* (planktonic foraminifer)

**DOI:** 10.1371/journal.pone.0191653

**Published:** 2018-01-29

**Authors:** Clare Bird, Kate F. Darling, Ann D. Russell, Jennifer S. Fehrenbacher, Catherine V. Davis, Andrew Free, Bryne T. Ngwenya

**Affiliations:** 1 School of Geosciences, University of Edinburgh, Edinburgh, United Kingdom; 2 School of Geography and Sustainable Development, University of St Andrews, St Andrews, United Kingdom; 3 Department of Earth and Planetary Sciences, University of California Davis, Davis, California, United States of America; 4 School of Biological Sciences, University of Edinburgh, Edinburgh, United Kingdom; Universita degli Studi di Urbino Carlo Bo, ITALY

## Abstract

Uncovering the complexities of trophic and metabolic interactions among microorganisms is essential for the understanding of marine biogeochemical cycling and modelling climate-driven ecosystem shifts. High-throughput DNA sequencing methods provide valuable tools for examining these complex interactions, although this remains challenging, as many microorganisms are difficult to isolate, identify and culture. We use two species of planktonic foraminifera from the climatically susceptible, palaeoceanographically important genus *Neogloboquadrina*, as ideal test microorganisms for the application of 16S rRNA gene metabarcoding. *Neogloboquadrina dutertrei* and *Neogloboquadrina incompta* were collected from the California Current and subjected to either 16S rRNA gene metabarcoding, fluorescence microscopy, or transmission electron microscopy (TEM) to investigate their species-specific trophic interactions and potential symbiotic associations. 53–99% of 16S rRNA gene sequences recovered from two specimens of *N*. *dutertrei* were assigned to a single operational taxonomic unit (OTU) from a chloroplast of the phylum Stramenopile. TEM observations confirmed the presence of numerous intact coccoid algae within the host cell, consistent with algal symbionts. Based on sequence data and observed ultrastructure, we taxonomically assign the putative algal symbionts to Pelagophyceae and not Chrysophyceae, as previously reported in this species. In addition, our data shows that *N*. *dutertrei* feeds on protists within particulate organic matter (POM), but not on bacteria as a major food source. In total contrast, of OTUs recovered from three *N*. *incompta* specimens, 83–95% were assigned to bacterial classes Alteromonadales and Vibrionales of the order Gammaproteobacteria. TEM demonstrates that these bacteria are a food source, not putative symbionts. Contrary to the current view that non-spinose foraminifera are predominantly herbivorous, neither *N*. *dutertrei* nor *N*. *incompta* contained significant numbers of phytoplankton OTUs. We present an alternative view of their trophic interactions and discuss these results within the context of modelling global planktonic foraminiferal abundances in response to high-latitude climate change.

## Introduction

Food web networks represent a range of pathways in ecological communities including predator-prey interactions, symbiotic associations, nutrient uptake and remineralisation that enable characterization of the transfer of nutrients and energy between species and trophic levels. This provides a basis for understanding large-scale ecosystem processes such as community structure and biogeochemical cycles. However, understanding these complex ecosystems is inherently limited by the methods available to study the links between predator and prey [1]. Whereas high-throughput DNA sequencing techniques have been adopted to address predator-prey interactions for many multicellular organisms [2–4], it is the viruses, prokaryotic and eukaryotic microorganisms that play fundamental roles within the food web and drive biogeochemical cycling [5–10]. To unravel the complexity of these marine microbial interactions, high-throughput sequencing of environmental DNA (eDNA) has been utilised to generate microbial association networks both at local (e.g. [11–13]) and global scales [14]. This holistic approach has uncovered previously unknown interactions between organisms, whether predator-prey, symbiont-host or parasitic associations, allowing targeted investigation of newly recognised interactions. Currently, such targeted investigations using high-throughput DNA sequencing methods of protists, their prey, and other interacting organisms are still in their infancy [15–17]. This is due to the difficulty in isolating, identifying and culturing specimens from this biogeochemically important group [16, 18]. Among protists, the planktonic foraminifera are comparatively easy to collect (plankton net tows and scuba diving) and isolate using stereo microscopy, due to their relatively large size (50–1000μm). They are also identifiable to the morphospecies and small subunit (SSU) ribosomal (r)RNA gene bar code level [19]. The morphology of their calcite shells and biogeographic ranges of their representative SSU rRNA genetic types are relatively well known [20]. They therefore represent an ideal organism for testing high-throughput DNA sequencing methodologies in the protists.

Understanding the ecology of planktonic foraminifera is particularly significant, as they are used extensively in the prediction of future climate change. The deposition and burial of their calcitic shells in the ocean sediments generates a fossil record that dates back 180 million years [21]. Their morphospecies assemblage composition and shell geochemistry in the sediments provides palaeoceanographers with numerous proxies for reconstructing past environmental conditions, which are used to constrain projections of future climate change [22–24]. Many proxies derived from the planktonic foraminiferal fossil record require species-specific calibration (e.g. [25–28]), due in part to the differing environmental preferences and ecology of the various species [29, 30]. Interpretation of the fossil archive therefore relies heavily on obtaining a thorough understanding of the ecology and biology of planktonic foraminifera in the water column of the modern ocean.

Ecological knowledge is also necessary for modelling the responses of planktonic foraminifers to changes in seawater temperature, stratification, pH and dissolved inorganic carbon (DIC) content as they respond to increasing anthropogenic CO_2_ levels [31, 32]. Such changes affect the rates of foraminiferal shell calcification [33, 34] which would modify calcite export from the surface and dissolution at depth, a cycle which buffers global ocean carbonate chemistry and atmospheric CO_2_ [33, 35–39]. Models currently suggest that non-spinose macro-perforate planktonic foraminifera of the genus *Neogloboquadrina* are most at risk from climate change [31, 32] in the order of increasing susceptibility from low-latitude to high-latitude species: *N*. *dutertrei<N*. *incompta<N*. *pachyderma*. This is not surprising given that high-latitude oceans have a naturally lower buffering capacity due to the higher solubility of CO_2_ in their cold waters (c.f. [38]). Arctic waters are therefore expected to be the first to experience under-saturation and dissolution of calcium carbonate minerals [40–43].

Currently, whilst the biogeographic distribution of the neogloboquadrinids is well known [44–48], knowledge of their ecology is limited to observations of a predominantly herbivorous diet and the inconsistent presence of putative algal symbionts in the lower latitude morphospecies, *N*. *dutertrei* [29]. These intact and abundant algae are thought to be of the class Chrysophyceae, (chrysophyte algae), due to their observed ultrastructure which includes the presence of a girdle lamella. They are also considered to be facultative rather than obligate symbionts [29, 30, 49–51] although a metabolic link is yet to be demonstrated. While the neogloboquadrinids are known to feed on unicellular algae [29, 52], there have been no investigations of the (trophic) interactions between the neogloboquadrinids and bacteria, which are highly abundant in the water column and in POM [53–55]. Such bacteria may therefore be a potential major food source for the neogloboquadrinids.

High-throughput DNA sequencing methods now permit an extensive examination of the algal and bacterial trophic profiles of the climatically susceptible and palaeoceanographically important genus *Neogloboquadrina*. In this study, we combine traditional observational methods (TEM and fluorescence microscopy) with 16S rRNA gene metabarcoding. This enables us to taxonomically identify the full range of bacterial/chloroplast sources of DNA from within single cells of the two *Neogloboquadrina* morphospecies *N*. *dutertrei* and *N*. *incompta* (previously *N*. *pachyderma* (dextral) [56]), collected at two sites off the coast of California. In this oceanographic region, only a single SSU rDNA genotype of each of *N*. *dutertrei* and *N*. *incompta* have been identified [56–58], *N*. *dutertrei* Type Ic and *N*. *incompta* Type II. We demonstrate that these genotypes have completely contrasting 16S rRNA gene sequence assemblages within their cells. With support from microscopy studies, we discuss the significance of their specific microbiotas and their trophic interactions for incorporation into ecological and modelling studies.

## Materials and methods

### Oceanographic setting, sample collection and preservation

The oceanographic setting off the coast of California and the location of sampling sites in this study are fully described in Bird et al. [59]. No specific permissions were required for collection at the chosen sampling locations, and no protected habitats or endangered species were involved. Details of sampling locations and processing information for collected individuals is listed in Table 1. Individual *N*. *dutertrei* were collected in July 2013 from a water depth of 40–50 m (water temperature at this depth ~11°C) via an open-close plankton net (Aquatic Research, 150μm mesh) offshore Santa Catalina Island (33.4° N, 118.4° W) in the San Pedro Basin, Southern California Bight. Tow material was transferred to ambient surface seawater and kept chilled during transit to shore at the Wrigley Marine Science Center, where live foraminifera were wet picked. Individual specimens of *N*. *dutertrei* were rinsed in 0.6 μm filtered surface seawater and preserved in RNA*Later*® (Ambion^TM^) for fluorescence microscopy and genetic analysis, or fixed in 3% glutaraldehyde for TEM. Individual specimens of *N*. *incompta* were collected along the narrow central California shelf between 1 and 32 km off Bodega Head, (38.3° N, 123.0° W) in April 2014 just after the onset of sustained, but weak, spring upwelling, [60] and during April-July 2015. Samples were obtained from vertically integrated 150μm mesh–size net tows, deployed to a maximum depth of 160 m, or to 10 m above the seafloor at shallower sites. Individual specimens of *N*. *incompta* were processed as described above and transferred to RNA*Later*® or 3% glutaraldehyde, at the Bodega Marine Laboratory. Sampling and processing information for collected individuals is listed in Table 1.

**Table 1:** Details of planktonic foraminiferal specimens collected.

Morphospecies	Sample ID	Sampling site	Sampling date	Co-ordinates	Sea surface temperature	Analysis
*N*. *dutertrei*	DUT41	Santa Catalina Island	July 2013	33.4°N, 118.4°W	18°C-21.5°C‡	Control for fluorescence microscopy
*N*. *dutertrei*	DUT43	Santa Catalina Island	July 2013	33.4°N, 118.4°W	18°C-21.5°C‡	DAPI staining
*N*. *dutertrei*	DUT44	Santa Catalina Island	July 2013	33.4°N, 118.4°W	18°C-21.5°C‡	DAPI staining
*N*. *dutertrei*	DUT45	Santa Catalina Island	July 2013	33.4°N, 118.4°W	18°C-21.5°C‡	DAPI staining
*N*. *dutertrei*	DUT46	Santa Catalina Island	July 2013	33.4°N, 118.4°W	18°C-21.5°C‡	DAPI staining
*N*. *dutertrei*	DUT47	Santa Catalina Island	July 2013	33.4°N, 118.4°W	18°C-21.5°C‡	DAPI staining
*N*. *dutertrei*	DUT48	Santa Catalina Island	July 2013	33.4°N, 118.4°W	18°C-21.5°C‡	DAPI staining
*N*. *dutertrei*	DUT49	Santa Catalina Island	July 2013	33.4°N, 118.4°W	18°C-21.5°C‡	Genotyping
*N*. *dutertrei*	DUT55	Santa Catalina Island	July 2013	33.4°N, 118.4°W	18°C-21.5°C‡	Metabarcoding and genotyping
*N*. *dutertrei*	DUT59	Santa Catalina Island	July 2013	33.4°N, 118.4°W	18°C-21.5°C‡	Metabarcoding
*N*. *dutertrei*	K129	Bodega Head	July 2015	38.3°N, 123.0°W	14°C-15°C	TEM
*G*. bulloides*	BUL34	Bodega Head	Nov 2014	38.3°N, 123.0°W	14°C-15°C	Metabarcoding and genotyping
*N*. *incompta*	INC25	Bodega Head	April 2014	38.3°N, 123.0°W	10°C-13°C	Metabarcoding
*N*. *incompta*	INC27	Bodega Head	April 2014	38.3°N, 123.0°W	10°C-13°C	Metabarcoding
*N*. *incompta*	INC28	Bodega Head	April 2014	38.3°N, 123.0°W	10°C-13°C	Metabarcoding
*N*. *incompta*	INC30	Bodega Head	April 2014	38.3°N, 123.0°W	10°C-13°C	DAPI staining
*N*. *incompta*	INC41	Bodega Head	April 2014	38.3°N, 123.0°W	10°C-13°C	TEM
*N*. *incompta*	INC42	Bodega Head	April 2014	38.3°N, 123.0°W	10°C-13°C	TEM
*N*. *incompta*	INC46	Bodega Head	April 2014	38.3°N, 123.0°W	10°C-13°C	DAPI staining
*N*. *incompta*	K100	Bodega Head	June 2015	38.3°N, 123.0°W	10°C-12°C†	TEM
*N*. *incompta*	K104	Bodega Head	June 2015	38.3°N, 123.0°W	10°C-12°C†	TEM
*N*. *incompta*	K105	Bodega Head	June 2015	38.3°N, 123.0°W	10°C-12°C†	TEM
*N*. *incompta*	K121	Bodega Head	July 2015	38.3°N, 123.0°W	14°C-15°C	TEM
*N*. *incompta*	K124	Bodega Head	July 2015	38.3°N, 123.0°W	14°C-15°C	TEM
*N*. *incompta*	K126	Bodega Head	July 2015	38.3°N, 123.0°W	14°C-15°C	TEM
*N*. *incompta*	F106	Bodega Head	July 2015	38.3°N, 123.0°W	14°C-15°C	TEM
*N*. *incompta*	F004	Bodega Head	April 2015	38.3°N, 123.0°W	10.5°C	TEM

Table 1 Individual samples collected from two sites offshore California with details of the sampling locations and processing information for each specimen collected for this study.

*This *G*. *bulloides* specimen is documented fully in Bird et al. [59]

‡ Temperature off Santa Catalina Island was obtained from the San Pedro Ocean Time Series data portal (https://dornsife.usc.edu/spot/cruise-log/)) for July 18, 2013

†Temperature at 1-m water depth obtained from Bodega Marine Lab obtained from Bodega Marine Lab Offshore Buoy located 1.2 km off Bodega Head at the 30-m isobath (http://boon.ucdavis.edu/bml_buoy.html)).

### Decalcification and washing of samples

Individual specimens for genetic analysis and fluorescence microscopy were decalcified during exposure to RNA*Later*® (Ambion^TM^), which dissolves the shell and removes shell–associated external contaminants [59]. Following decalcification, the naked cell was then washed in filter-sterilised, salt-adjusted phosphate buffered saline (PBS) or sterile artificial seawater and transferred to a sterile 1.5 ml tube. The cell washing procedure was repeated three more times and the cell was then transferred into DOC DNA extraction buffer [61] for DNA analysis, or 4% (w/v) paraformaldehyde in salt–adjusted PBS for fluorescence microscopy.

### Foraminifera genotyping and Sanger DNA sequencing

DNA was extracted from individual foraminifer specimens using the DOC extraction method [61] for partial SSU rRNA gene amplification to identify the specific genotype. PCR was performed according to Seears et al. [62]. PCR products were ligated into the pGEM®-T Easy Vector (Promega) and transformations were carried out in JM109 (Promega) competent cells according to the manufacturer’s protocol. DNA sequencing was carried out using the BigDye® Terminator v3.1 Cycle Sequencing Kit and an ABI 3730 DNA sequencer (both Applied Biosystems).

### DNA extraction, amplification and 16S rRNA gene metabarcoding

DNA for 16S rRNA gene metabarcoding was extracted from the decalcified and washed cells using the DOC extraction method [61] The DNA from five Neogloboquadrina specimens were amplified together with three reagent controls as follows: *N*. *dutertrei* (DUT55 and DUT59) from Santa Catalina Island, *N*. *incompta* (INC25, INC27 and INC28) from Bodega Head, 2 x Controls with no DNA template and 1 x Control with DOC buffer only. The V4 region of the 16S rRNA gene was chosen for amplification using the 515F forward primer and a barcoded 806R reverse primer series [63]. This primer set amplifies a 253bp DNA fragment. DNA degradation in prey items limits the success of amplification of DNA sequences greater than ~250bp [2]. Therefore this primer set provides information not only about intact undigested bacteria and chloroplasts, but also about those phagocytosed for food. These primers are widely used by the Earth Microbiome Project [64] and therefore the amplification biases are known and well documented. For example, there is a bias against amplification of the SAR11 group of marine Alphaproteobacteria, and a slight bias towards over amplification of Gammaproteobacteria [65–67]. The thermal cycling conditions are detailed by Caporaso et al., [63] and PCR reactions described by Bird et al., [59]. Next-generation DNA sequencing was performed at Edinburgh Genomics using an Illumina MiSeq v2 to generate 250 base pair (bp) paired-end reads.

### Quality filtering, operational taxonomic unit (OTU) picking, and taxonomic assignment

The Quantitative Insights in Microbial Ecology (QIIME, v1.8.0, [68]) pipeline was used to assemble paired–end reads and quality filter the sequences as described by Bird et al., [59]. Chimeras were detected using Usearch v6.1.544 default settings [69] and version 13_8 of the Greengenes 16S rRNA gene references database [70]. The default QIIME pipeline was used for both de novo OTU picking and closed reference OTU picking followed by taxonomic assignment also using version 13_8 of the Greengenes 16S rRNA gene database [70]. De novo picking clusters DNA sequences into OTUs with 97% similarity with no external reference and selects a representative sequence of each OTU for alignment and subsequent assignment of taxonomy. This script keeps all diversity, including unknowns in the sample set. Closed reference picking was also performed which removes OTUs that are not closely matched (<97%) with OTUs in the Greengenes database. This output is required for Normalisation By Copy Number, developed for the PICRUSt pipeline [71] using the online Galaxy tool (http://huttenhower.sph.harvard.edu/galaxy/). This corrects the abundance of each OTU to better reflect the true organism abundance by normalising predicted 16S rRNA gene copy number for each OTU. In both OTU picking methods, OTUs with fewer than 10 sequences across all samples were removed from the sample set.

### Contaminant removal

Given the low yield of endogenous bacterial DNA in these small-sized samples, it was anticipated that amplicon contamination from PCR amplification reagents, DNA extraction reagents, and the ultra-pure water system would contribute a significant number of DNA sequences and OTUs from contaminant genera to the sample set [72, 73]. Contaminant OTUs were removed according to Bird et al. [59]. Two OTUs were removed due to contamination in the two PCR controls; a Bradyrhizobiaceae OTU of the class Alphaproteobacteria and an *Acinetobacter* OTU of the class Gammaproteobacteria. Ten contaminating OTUs were removed due to contamination via the DOC buffer, six of these were also of the class Alphaproteobacteria, order Rhizobiales, with four classified to a lower taxonomic rank including a second Bradyrhizobiaceae; a *Methylobacterium*; a *Mesorhizobium*; and a *Pedomicrobium*. One final Alphaproteobacteria OTU was removed, a *Sphingomonas* of the order *Sphingomonadales*. The final three OTUs were *Burkholdaria bryophila* of the class Betaproteobacteria; *Sediminibacterium* of the phylum Bacteriodetes; and a chloroplast OTU of the Streptophyta. A single Bradyrhizobiaceae OTU was by far the largest contaminant with a total of 130,244 sequences from the three control samples (224,399 sequences across all samples) and it is known to be, together with other Alphaproteobacteria, a common contaminant of next-generation sequencing data [73].

### Alpha-rarefaction and sequencing depth

In QIIME, the script alpha_rarefaction.py was used to assess whether the sequencing depth (i.e. the numbers of sequences generated for each sample) was adequate to detect the full range of bacterial diversity found in each foraminiferal specimen. Samples were rarefied to the lowest sequencing depth observed across all samples (57,929 sequences in closed reference picking and 57,177 sequences in de novo picking, both in DUT59). Rarefaction curves for OTU richness (S1 Fig) were generated using the observed species metric which counts the number of unique OTUs found in a sample. The numbers of new OTUs increased rapidly up to 6,000 sequences per sample (demonstrated by the steepness of the curve), before slowing (demonstrated by flattening curves), confirming that the sequencing depth was sufficient to capture the full bacterial assemblage diversity within each species.

### Fluorescence and transmission electron microscopy

Individual *N*. *dutertrei* (n = 6) and *N*. *incompta* (n = 2) cells were stained with 4’,6–diamadino–2–phenylindole (DAPI) following the procedure of Bird et al. [59]. Exposure of cells to DAPI causes the formation of a highly fluorescent DAPI–DNA complex that allows the visualisation of bacterial cells and eukaryotic cell nuclei under fluorescence microscopy. An unstained specimen of *N*. *dutertrei* (Table 1, DUT41) was also examined by fluorescence microscopy to observe the background levels of autofluorescence under the DAPI filter set to compare with the appearance of DAPI-stained individuals.

Transmission Electron Microscopy (TEM) was used to observe and document the structural relationships between the internal microorganisms and foraminiferal cells. *N*. *dutertrei* and *N*. *incompta* were fixed following the protocol of Spero [74] to decalcify the foraminifera after initial fixation in 3% glutaraldehyde. Ultrathin sections (60 nm) were cut from selected areas, stained in Uranyl Acetate and Lead Citrate, and then viewed in a JEOL JEM-1400 Plus transmission electron microscope.

## Results

In total, 26 specimens of the two planktonic foraminiferal morphospecies *N*. *dutertrei* (n = 11) and *N*. *incompta* (n = 15), collected off Santa Catalina Island and Bodega Head, were investigated during this study. The sampling information is shown in Table 1 and the sampling strategy and genetic characterisation is described in the methods.

### Genetic characterisation

The partial SSU rRNA gene sequences amplified from specimens DUT55 and DUT49 identified them as *N*. *dutertrei* Type Ic. The DUT55 SSU DNA sequence (1000 bp) was submitted to Genbank (NCBI, accession number KX816048 [59]. This genotype has been found routinely in the Southern California Bight [57] and no other genotype has been reported in the region. Therefore, we are confident that all the individuals analysed in this study were *N*. *dutertrei* Type Ic. Although the amplification of the SSU rRNA gene of *N*. *incompta* proved unsuccessful, we are also confident that it was *N*. *incompta* Type II. Only two *N*. *incompta* genotypes have been identified globally (Types I and II). Type I has been found throughout the North and South Atlantic, while only Type II has been identified within the northeast Pacific waters of the California Current [21, 57, 58], within our study area.

### 16S rRNA gene metabarcoding

16S rRNA gene metabarcoding was carried out on two specimens of *N*. *dutertrei* (DUT55, DUT59) and three specimens of *N*. *incompta* (INC25, INC27, INC28). This raw dataset is submitted to the sequencing read archive (SRA, NCBI); Bioproject accession PRJNA341096, Biosample accessions SAMN07249166-SAMN07249169, SRA run accessions SRR5710159-SRR5710162. A total of 1,226,456 sequences were generated by Illumina sequencing from the five samples and three controls after quality filtering. After removing control sequences and control contaminant OTUs from the dataset, a total of 741,768 sequences (closed reference picking) and 742,871 sequences (de novo picking) were clustered in OTUs and taxonomically assigned. The numbers of sequences and OTUs generated in individual specimens for both closed reference picking and de novo picking are shown in S1 Table. Since the within-specimen OTU profiles were highly comparable between de novo picking and closed reference picking with normalisation by copy number, we present results for closed reference picking with normalisation by copy number.

**Fig 1:**
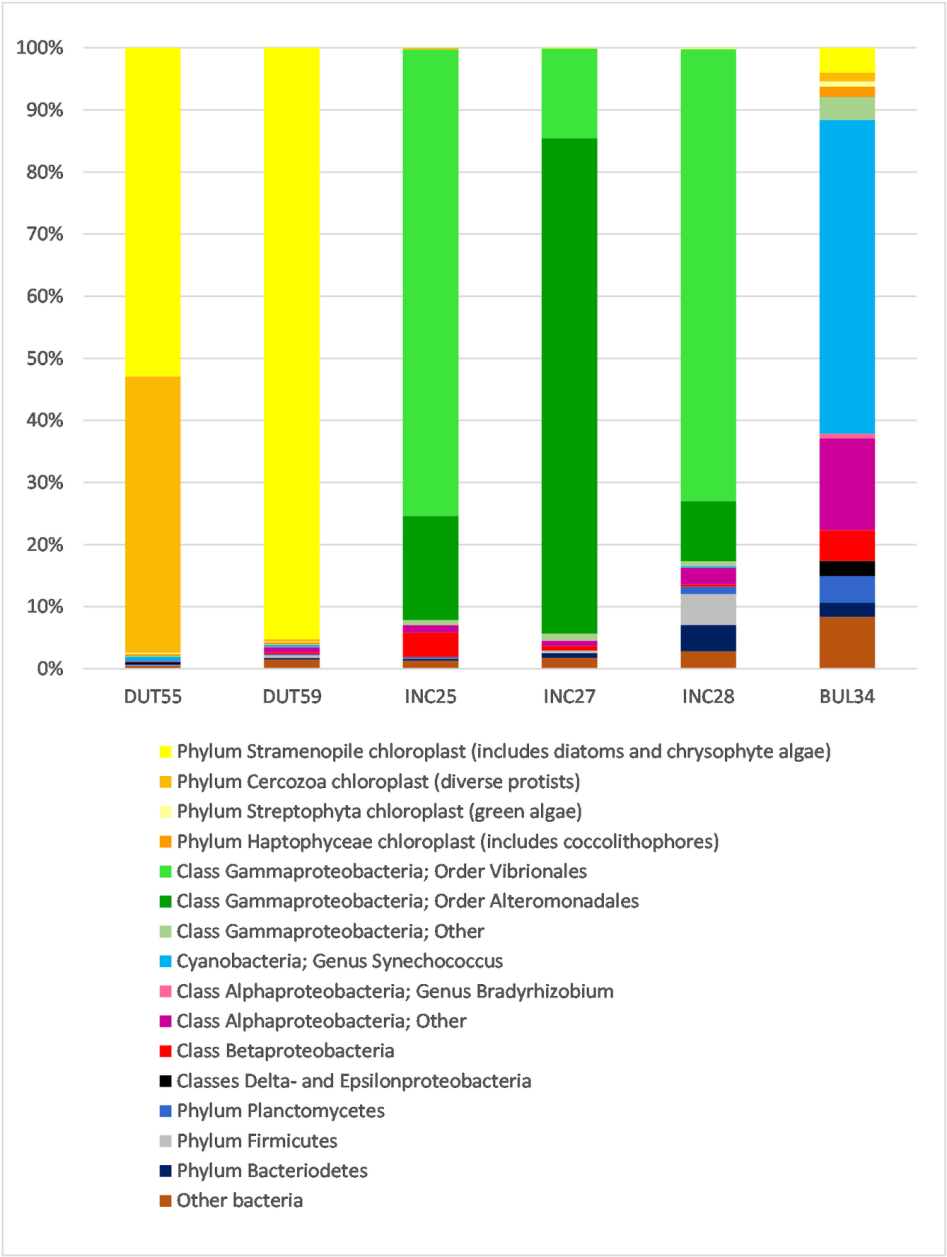
Relative abundance of taxonomically assigned 16S rRNA gene sequences from six individual foraminifer specimens. Two *N*. *dutertrei* (DUT55 and DUT59), three *N*. *incompta* specimens (INC25, INC27 and INC28) and one *G*. *bulloides* specimen (BUL34; [59]) are shown for comparison. Sequences are assigned to operational taxonomic units (OTUs) and have been grouped in the figure at higher levels of taxonomic classification for visual clarity (see key). Assignation of lower taxonomic ranks are discussed in the text, such as the OTUs grouped within the Gammaproteobacteria, in the order Vibrionales.

The taxonomic composition and relative abundance of OTUs in each of the *Neogloboquadrina* specimens is shown in Fig 1. An individual specimen of the spinose planktonic foraminifera, *Globigerina bulloides*, (BUL34, Table 1; [59]) is also shown for additional comparison. All three morphospecies contain a substantially different 16S rRNA gene assemblage, with a high degree of consistency in the microbial assemblage between individuals of the same species. However, there are differences in the relative composition of the microbial populations between specimens.

### 16S rRNA gene assemblage in *N*. *dutertrei*

Two individual *N*. *dutertrei* (DUT55 and DUT59; Table 1) were investigated (Fig 1). In the first individual (DUT55), 53% of all sequences are assigned to nine Stramenopile chloroplast OTUs (phylum containing diatoms and Chrysophyceae). However, the majority of these Stramenopile chloroplast sequences (99%) are found within a single OTU, the representative sequence of which in turn has a 99% match to algae of the class Pelagophyceae (GenBank accession LN735509, a sister class to the Chrysophyceae). In addition, 45% of sequences in DUT55 are assigned to four Cercozoa (phylum of mixotrophic protists) chloroplast OTUs, of which one OTU is dominant (92% of Cercozoa sequences). In DUT55, Stramenopile and Cercozoa sequences together account for over 98% of the sequence assemblage. In the second individual (DUT59), Stramenopile chloroplasts contribute more than 95% of sequences across only three OTUs, with 99.9% of all Stramenopile sequences belonging to the same single OTU as that dominating in DUT55, and related to the Pelagophyceae. Whilst this second individual does contain two Cercozoa chloroplast OTUs, the relative abundance of these sequences is only 0.4%. In both cases, bacterial sequences contribute very little: 2%–4% of the assemblage.

### 16S rRNA gene assemblage in *N*. *incompta*

The three *N*. *incompta* replicates, (INC25, INC27 and INC28; Table 1) contain the larger numbers of OTUs (S1 Table; S1 Fig) of the two morphospecies investigated in this study. Despite the relatively high diversity observed, there is a striking similarity in the bacterial assemblage across the replicates. 93%, 95% and 83% of all sequences in INC25, INC27 and INC28 respectively are of the class Gammaproteobacteria (Fig 1). There are 14 orders within the class Gammaproteobacteria, nine of which are represented within the *N*. *incompta* specimens contributing to 131 OTUs in total. Just two of these orders, however, contain the majority of the Gammaproteobacteria sequences; the Alteromonadales which contribute 73 OTUs and the Vibrionales which contribute 32 OTUs. The other seven orders each generally contribute less than 0.5% sequence abundance across 26 OTUs.

Specimens INC25 and INC28 contain more Vibrionales (75% and 73% respectively) compared to Alteromonadales sequences (17% and 10% respectively). All of the Vibrionales are of the family Vibrionaceae with sequence abundances of 67% (INC25), 14% (INC27) and 24% (INC28) found within 15 OTUs classified only to this level. However, four OTUs of the family Vibrionaceae were assigned to the genus *Allivibrio* and comprise sequences abundances as high as 40% in INC28 with 2.4% in INC25 and less than 1% in INC27. 12 OTUs assigned to the genus *Photobacterium* make up 3% (INC25) 0.1% (INC27) and 4% (INC28) of sequence abundance.

INC27 differs from INC25 and INC28 in that it contains fewer Vibrionales than Alteromonadales sequences; 14% Vibrionales described above, and 80% Altermonadales. The main contributor to the order Alteromonadales bacterial assemblage in INC27 are 32 OTUs of the family Pseudoalteromonadaceae which together make up 46% of all sequences in this specimen. Two further groups identified to the family level are also of import; Colwelliaceae (19% across 13 OTUs) and Alteromonadaceae (12% across 7 OTUs).

All *N*. *incompta* specimens contain negligible chloroplast 16S rRNA gene OTUs, with less than 0.2% of sequences assigned to 10 OTUs from a range of phyla (Stramenopiles, Cercozoa, Streptophyta and Haptophyta).

Both neogloboquadrinids also differ from *G*. *bulloides* Type IId (Fig 1; [59]). Aside from the large proportion of *Synechococcus* currently only observed in this genotype, *G*. *bulloides* contains negligible numbers of Alteromonadales and Vibrionales, instead containing a range of OTUs assigned to the Alphaproteobacteria. *G*. *bulloides* also contains few chloroplast OTUs.

### Fluorescence and transmission electron microscopy

Targeting a short 253 bp DNA fragment in 16S rRNA gene metabarcoding enables amplification of the degraded DNA of prey items [2] but will also amplify the intact DNA of any endobionts/symbionts housed within the foraminiferal cell. To determine whether any of the 16S rRNA genes, sequenced and taxonomically assigned in the metabarcoding performed in this study were from endobionts/putative symbionts rather than from prey organisms, microscopy was used to observe and document the structural relationships of any intact and more broadly distributed algal/bacterial cells within the host cytoplasm (e.g. [47, 59, 75]).

### Fluorescence microscopy of *N*. *dutertrei*

**Fig 2:**
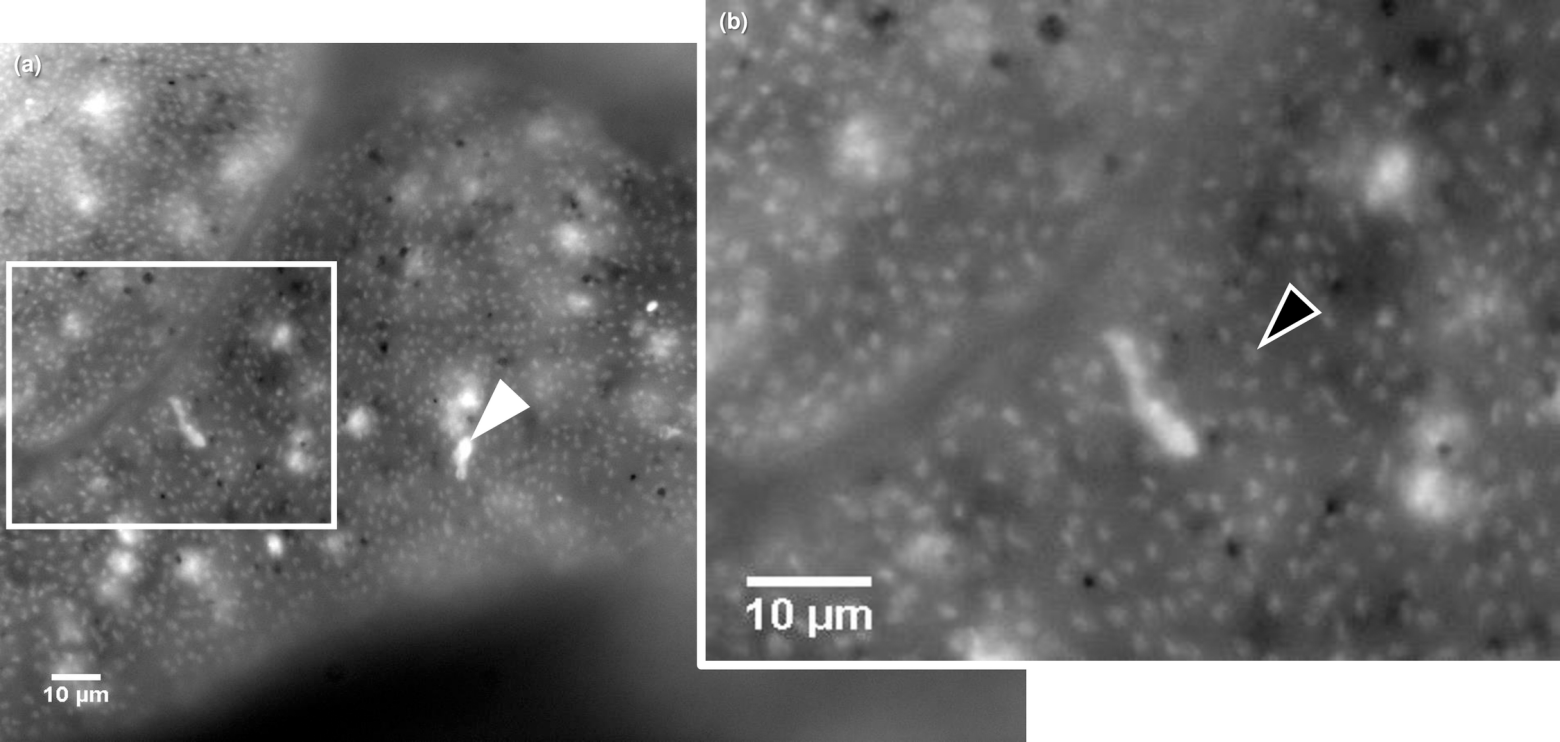
Fluorescence micrograph of a DAPI-stained decalcified *N*. *dutertrei* cell. (a) Diffuse autofluorescence can be observed throughout the cytoplasm. The observed ~2 μm diameter structures prevalent throughout the cell are consistent with the presence of picoeukaryotic algae. The white arrowhead denotes an example of the bright spots, 5–10μm in size that may be food vacuoles containing condensed prey items. The white rectangle denotes the area magnified in (b) where the black arrowhead highlights one of the ~2 μm diameter putative picoeukaryotes.

**Fig 3:**
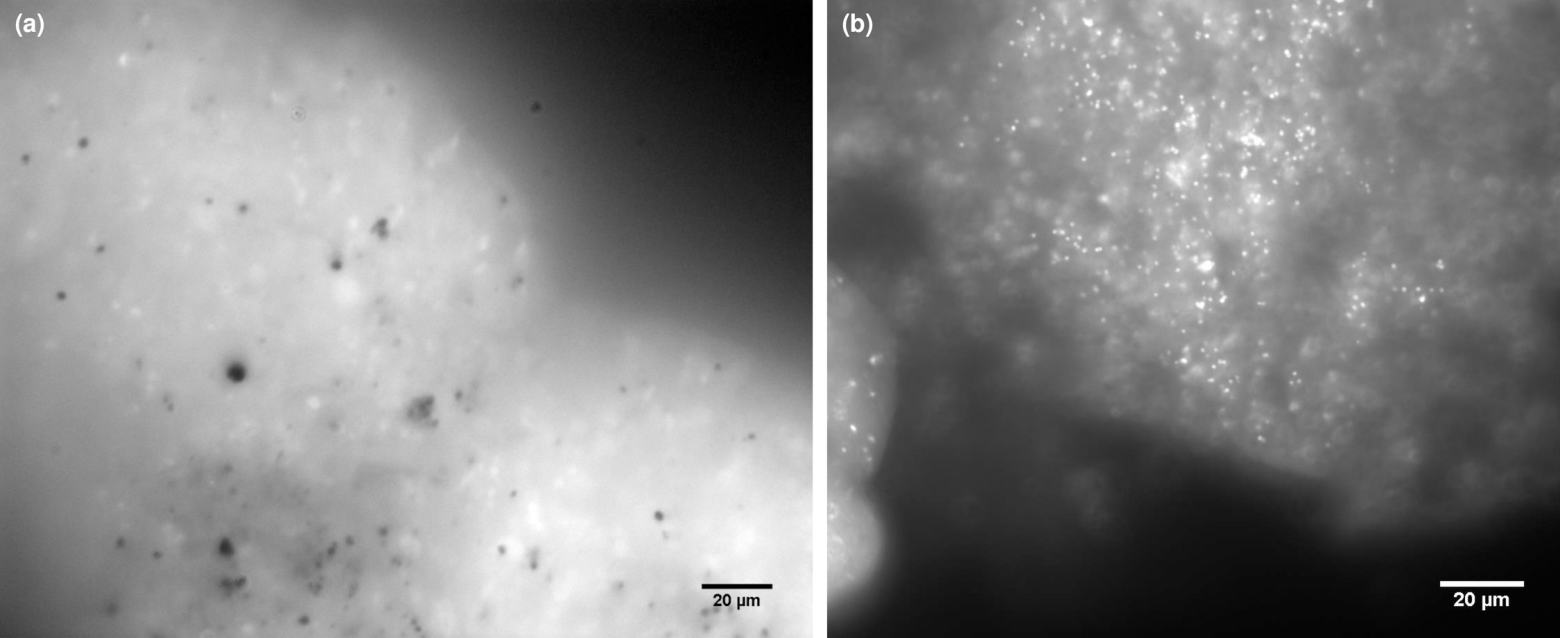
Fluorescence micrographs of decalcified *N*. *incompta* and *G*. *bulloides* cells. (a) High levels of background autofluorescence can be observed throughout the cytoplasm of the DAPI-stained decalcified *N*. *incompta* cell. However, in contrast to *N*. *dutertrei* (Fig 2), there are no abundant algal nuclei observable above background signals. In addition, in contrast to decalcified *G*. *bulloides*, where cyanobacterial cells can easily be detected (b) no bacterial cells can be observed in *N*. *incompta*.

**Fig 4:**
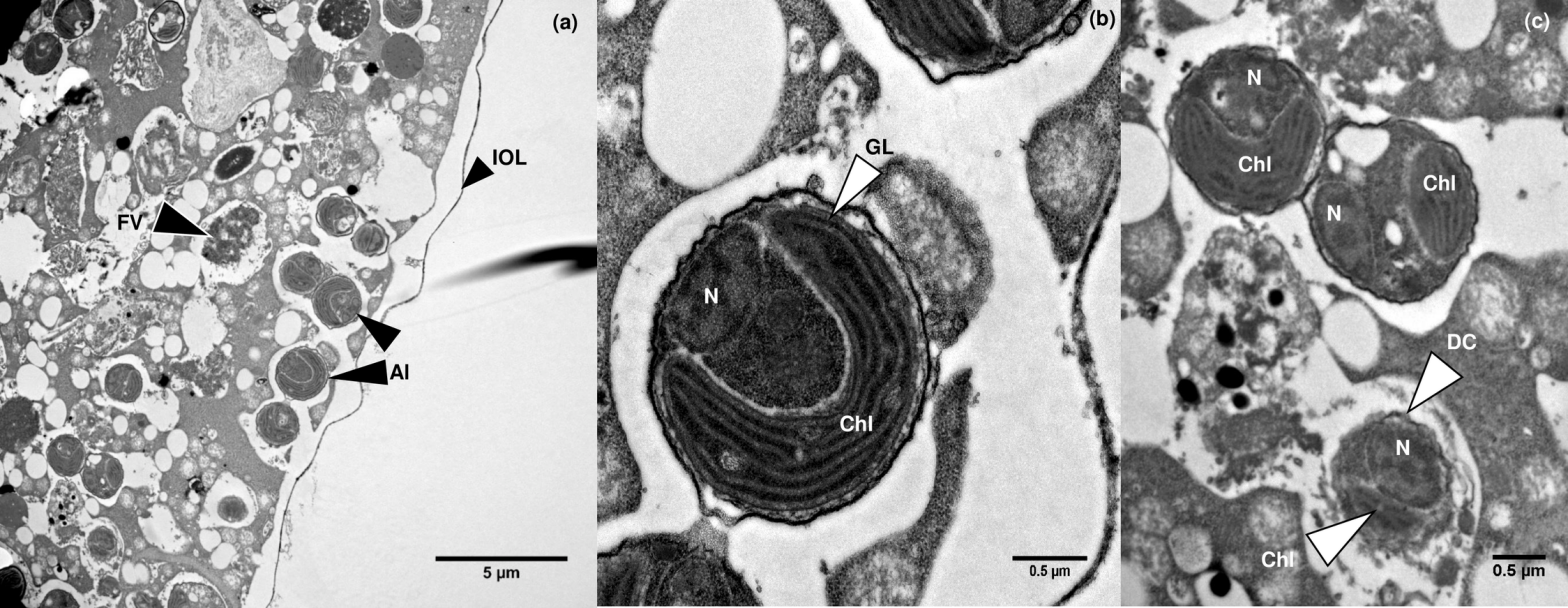
Transmission electron micrographs of pelagophyte cells inside *N*. *dutertrei*. (a) Intact picoeukaryotic algae can be observed (Al) in close proximity to the cell periphery. The inner organic layer (IOL) is also clearly visible. A probable food vacuole can also be seen (Fv). (b) An individual algal cell with a clearly visible horse-shoe shaped chloroplast (Chl) with girdle lamella (GL) and discernible nucleus (N). (c) Two intact algal cells with obvious chloroplasts and nuclei can be seen above an algal cell being digested (DC). The nucleus and chloroplast of this cell are still observable, but the cell membrane appears no longer to be fully intact.

Examination of an unstained, fixed *N*. *dutertrei* specimen (DUT41; Table 1) by fluorescence microscopy demonstrates high levels of diffuse autofluorescence across the entire cell. However, in all DAPI-stained *N*. *dutertrei* cells (n = 6; Table 1) a uniform arrangement of large numbers of nuclei across the cell was observed above background autofluorescence (Fig 2). These fluorescing structures are of a size (~2 μm) comparable to the algal symbionts found within the cytoplasm of the spinose planktonic foraminiferal species *Globigerinella siphonifera* Type II [50] and to those reported in *N*. *dutertrei* [49]. This observation agrees with 16S rRNA gene metabarcoding which assigned 53% (DUT55) and 95% (DUT59) of OTUs to a Stramenopile source. DAPI staining also indicates that there are no identifiable bacterial cells within the *N*. *dutertrei* cell. Bacteria would have a fluorescent signature of less than 1 μm, as seen in fluorescence micrographs of *G*. *bulloides* cells which contain bacterial endobionts (for example, see Fig 3; [59]). Again, these data support the findings of 16S rRNA gene metabarcoding which assigned only 2%–4% of sequences to bacterial OTUs. In addition, in DAPI-stained *N*. *dutertrei* cells some highly fluorescent regions of 5–10 μm diameter can be observed (Fig 2). These stained structures are similar in size range to food vacuoles (for example, see Fig 4) and therefore are likely to be DAPI–DNA complexes in organisms sequestered within food vacuoles.

### Fluorescence microscopy of *N*. *incompta*

DAPI-stained *N*. *incompta* cells express high levels of background autofluorescence (Fig 3A). In contrast to *N*. *dutertrei* (Fig 2), no algal nuclei can be observed. This observation is consistent with 16S rRNA gene metabarcoding, which assigned < 0.2% of sequences to a chloroplast source in this species. However, despite > 99% of all sequences being assigned to bacterial OTUs in the 16S rRNA metabarcoding of *N*. *incompta*, intact bacterial endobionts are not observed (Fig 3A), as they were in the *G*. *bulloides* cell (Fig 3B; [57]). This suggests that bacteria are solely prey organisms in *N*. *incompta*, and that there are no endobiotic/symbiotic associations. Some brighter regions of fluorescence within the DAPI-stained *N*. *incompta* cells are of a similar size to those in *N*. *dutertrei* (5–10 μm) and hence may also be DAPI–DNA complexes in organisms sequestered within food vacuoles.

### TEM of *N*. *dutertrei*

Fluorescence microscopy identified a wealth of picoeukaryotes evenly distributed within the *N*. *dutertrei* cell (Fig 2). TEM was therefore used to observe and document the structural relationship between these putative symbionts and the *N*. *dutertrei* cell. Numerous algal cells were observed within the single specimen of *N*. *dutertrei* investigated (K129; Table 1), many of which were found close to the host cell membrane (Fig 4A). The cells have a distinctly coccoid appearance and contain a single large horseshoe-shaped chloroplast with a girdle lamella, characteristic of the Pelagophyceae (Fig 4A; [76]). However, small numbers of algae appeared to be in a state of digestion within the host cell (Fig 4C). No other cell types were observed within the foraminiferal cell, which might indicate that the sequences assigned to Cercozoa via 16S rRNA gene metabarcoding in DUT55 were derived from a food source, although analysis of further samples is required to confirm this. No bacterial cells were observed in this specimen.

### TEM of *N*. *incompta*

**Fig 5:**
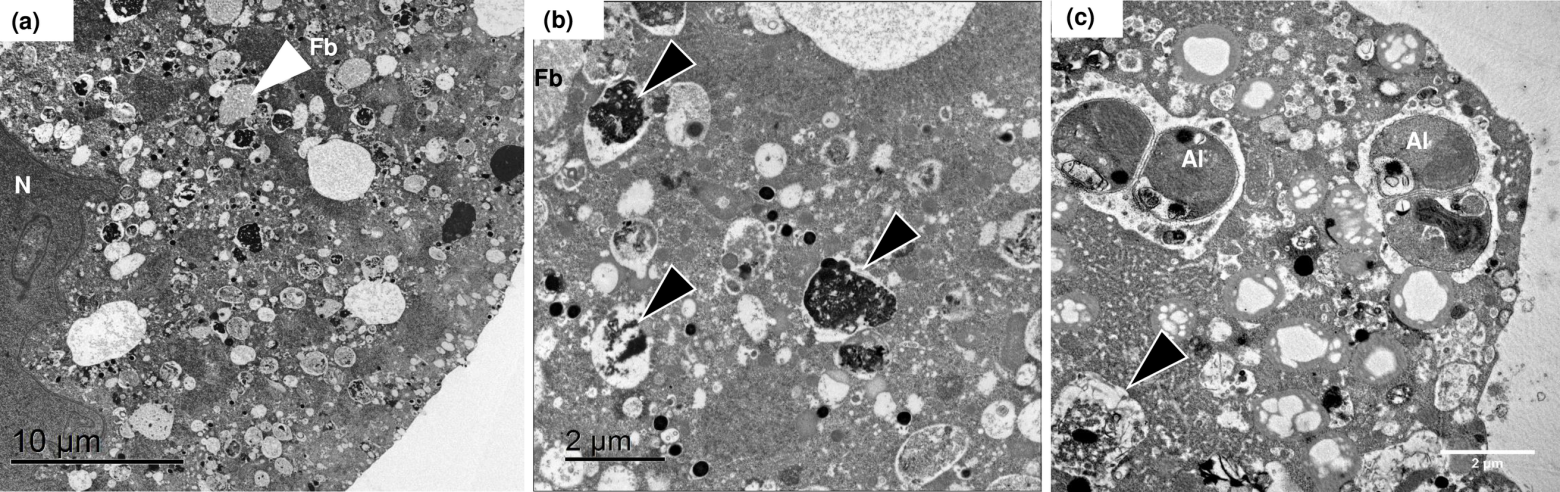
Transmission electron micrographs of *N*. *incompta*. (a) Low-magnification region of *N*. *incompta* showing the nucleus (N) and a fibrillar body (fb). (b) Higher magnification image shows a fibrillar body (Fb) and probable food vacuoles (black arrowheads). (c) A very small number of algal cells in early degradation (Al) were observed in some *N*. *incompta* individuals.

**Fig 6:**
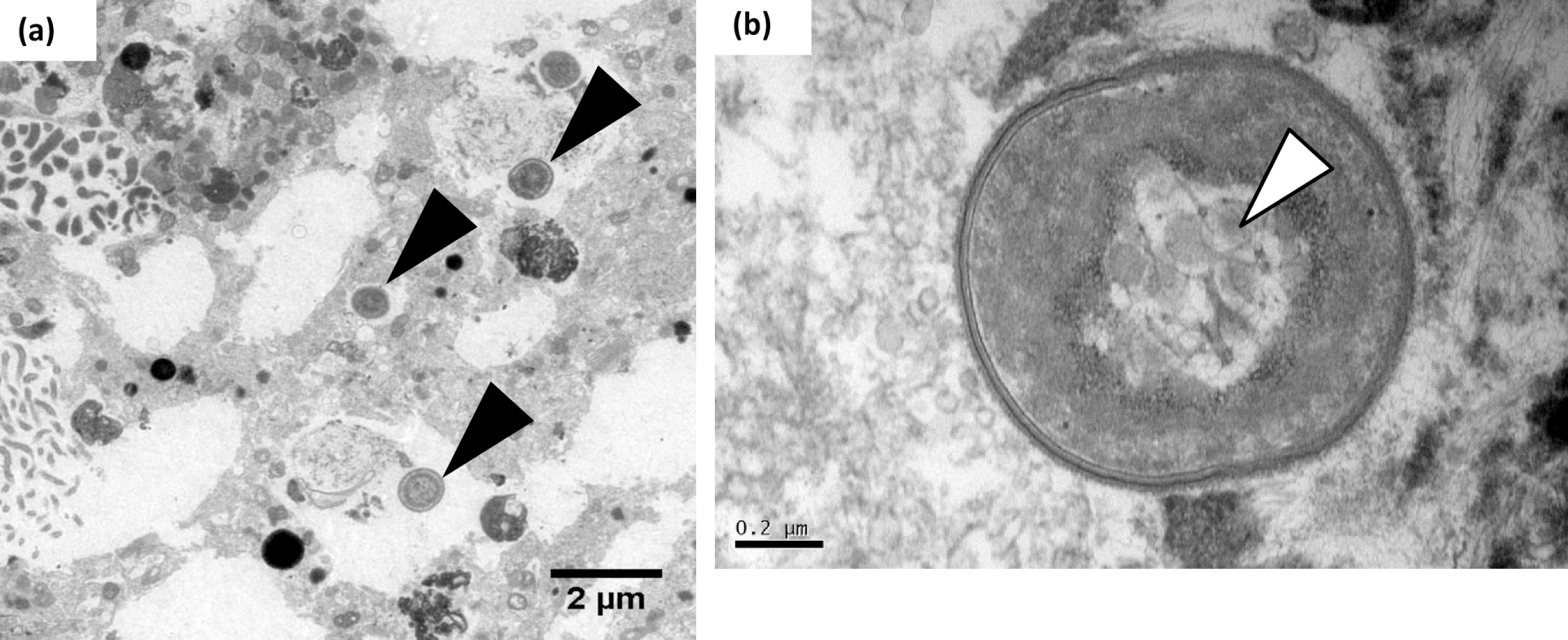
Transmission electron micrographs of *G*. *bulloides*. (a) The black arrowheads depict cyanobacteria of the genus *Synechococcus* [59] within the *G*. *bulloides* cytoplasm. (b) Higher magnification of a *Synechococcus* endobiont, with visible characteristic carboxysomes (white arrowhead).

TEM imaging was performed on ten *N*. *incompta* specimens (Table 1) to investigate whether bacteria of the orders Vibrionales and Alteromonadales identified via 16S rRNA gene metabarcoding (83%–95% of sequences; Fig 1) were observable in the foraminiferal cell. Despite the numerous vesicles of < 1 μm present in the *N*. *incompta* micrographs, none were bound by a cell membrane and hence no bacterial endobionts were observed in the cell (Fig 5A and 5B). Unlike in *G*. *bulloides* (See Fig 6; [59]), there was no bacterial population with consistent morphology, position or abundance in any of the *N*. *incompta* individuals examined. Of note is the presence of a small number of algal cells in all the *N*. *incompta* specimens, whose cytoplasm is undergoing degradation (Fig 5C) unlike those observed in *N*. *dutertrei* (Fig 4B). Their extremely low numbers are indicative of a limited food source rather than of a symbiotic relationship. This is supported by the 16S rRNA gene metabarcoding data, which documents just 0.2% of sequences corresponding to chloroplasts from the phylum Cercozoa (mixotrophic protists; INC25), 0.1% of sequences assigned to chloroplasts from Stramenopiles (includes diatoms, Chrysophyceae and Pelagophyceae; INC27) and 0.2% of sequences corresponding to chloroplasts of Streptophyta (includes green algae; INC28).

## Discussion

*N*. *dutertrei* and *N*. *incompta* are non-spinose macro-perforate planktonic foraminifera that are often, but not exclusively, found within an aggregation of POM (personal observations) which, we suggest, is a feeding cyst. Feeding cysts are extremely common in many species of benthic foraminifera [77, 78]. They have also been reported in association with the non-spinose micro-perforate planktonic foraminifera *Globigerinita glutinata* [79], where a TEM image shows the feeding cyst to have a structured cyst wall, suggesting it was not a sampling artefact. In addition, the geochemical signatures (for Ba, Mn, Cd, Zn) of deeper dwelling non-spinose planktonic foraminifera provide evidence that they are not calcifying in open seawater [80]. If this is the case, a POM microhabitat may be an alternative calcifying environment. The current literature indicates that *N*. *dutertrei* and *N*. *incompta* are predominantly herbivorous [29], but our data suggest that these two species have evolved contrasting ecological strategies. *N*. *dutertrei* Type Ic contains significant numbers of intact intracellular algae likely to be symbionts, and is a predator of protists and not of the bacteria colonising POM. In contrast, our data demonstrate that *N*. *incompta* Type II feeds much less frequently on phytoplankton than previously considered, and that Gammaproteobacteria of the orders Vibrionales and Alteromonadales make up a significant component of its diet. Below we discuss the evidence for these contrasting ecological strategies, and the value in understanding their trophic interactions for ecological and modelling studies.

### Trophic interactions of *N*. *dutertrei*

#### Taxonomic classification of putative algal symbionts as Pelagophyceae

From the 16S rRNA gene metabarcoding, fluorescence microscopy and TEM data, we suggest that the ecological strategy of *N*. *dutertrei* Type Ic includes a symbiotic association with a single species of algae from the phylum Stramenopile. This phylum includes both the diatoms and the class Chrysophyceae. Previous workers have proposed that the picoeukaryotic putative symbionts observed in *N*. *dutertrei* and a number of other planktonic foraminiferal species are chrysophytes, based on their investigations of ultra-structure [49, 50]. However, a GenBank BLAST search, using the representative sequence for the abundant Stramenopile OTU from our dataset, returned a 99% match with algae of the class Pelagophyceae [76], rather than the class Chrysophyceae. The Pelagophyceae are coccoid marine algae identified globally, including in the Pacific Ocean and the Southern California Bight [81, 82]. In the Southern California Bight, *N*. *dutertrei* is found predominantly above 100 m throughout the year, with peak population abundance above 50 m in spring and early summer [83–85], i.e. in the photic zone. This also coincides well with the depth profile of Pelagophyceae (preference for 0–50 m with a maximum depth of 90 m; [81]http://www.eol.org/pages/3530/overview). Like species in the class Chrysophyceae, Pelagophyceae contain a single chloroplast with girdle lamella [76], as can be seen in Fig 4B. Therefore, based on the 16S rRNA gene metabarcoding, and TEM evidence, we propose that the putative symbionts of *N*. *dutertrei* Type Ic are Pelagophyceae, a class currently containing 16 species in 13 genera [86], and suggest that this marine class may be found in a range of other planktonic foraminifera species originally thought to contain Chrysophyceae. For example, *G*. *siphonifera* Type I and II contain two different coccoid symbionts [50]. The Type I symbionts are genetically characterised as a prymnesiophyte of the haptophyte lineage [87]. However, Type II symbionts have not yet been genetically characterised and Gast et al. [87] suggest that these symbionts might be a chrysophyte algae, as was also proposed for *N*. *dutertrei* by Gastrich [49]. Additional DNA sequencing of the 18S rRNA genes of the symbiont and its foraminiferal hosts will provide further information on both the classification of the symbiont and the foraminiferal morphospecies and genotypes that contain it.

#### The host-algae relationship

We consider that the Pelagophyceae in *N*. *dutertrei* Type Ic are highly likely to be symbionts for two reasons. Firstly, fluorescence microscopy examination (Fig 2) and TEM imaging (Fig 4) demonstrate a well-ordered distribution pattern, with many cells in close proximity to the foraminiferal cell membrane. This pattern, also observed by Hemleben et al. [29], would optimise light access for photosynthesis and streaming along the rhizopodial network (see below). Secondly, such coccoid algae have been identified in a range of foraminifera and are reported as (facultative) symbionts [29, 49–51, 87–89]. The putative symbionts of *N*. *dutertrei* are amongst those labelled as facultative, because Hemleben et al. [29] reported that some individual *N*. *dutertrei* cells lacked algae, and because he observed some algae in a state of digestion. All the *N*. *dutertrei*, collected during the summer in this study (n = 11), and all samples (n = 22) observed by Gastrich [49] in the autumn and winter months from the Atlantic Ocean, contained living algae. Therefore, unlike in *Globorotalia hirsuta*, which lacks symbionts in the winter months [49], seasonality is not likely to be the reason for the absence of putative symbionts in *N*. *dutertrei*. Observations at varying stages of ontogeny may be a factor in the presence or absence of algae. For example, juvenile *N*. *dutertrei* are thought to dwell higher in the water column than adults, where the light regime would benefit a symbiotic association. Adults sink from the surface waters, calcifying at depth prior to gametogenesis [90]. It is possible that juveniles have an association with these putative symbionts to facilitate their growth and development into adults, and that the algae are then lost, possibly via digestion, as the adults sink and become gametogenic. This theory is supported by laboratory observation that symbionts are either expelled from the cell (personal observations J. Fehrenbacher) or are digested [29, 91] just prior to gametogenesis. Finally, the limited observations of *N*. *dutertrei* lacking putative algal symbionts may be a function of differing strategies between various genotypes.

The nature of the relationship between *N*. *dutertrei* Type Ic and its putative pelagophyte algal symbiont requires further examination. Evidence that some of these algae are found in a state of digestion (this study, [29]) does lend some support to a facultative symbiotic association. However, in both *G*. *siphonifera* Type I with obligate prymnesiophyte symbionts and *G*. *siphonifera* type II with obligate, possible chrysophyte [87] or pelagophyte algal symbionts, some digestion of symbionts does occur throughout the life time of the host, and therefore this cannot be considered a trait only of facultative symbiont-host relationships. For example, although unlikely given our understanding of the dinoflagellate symbiotic system [92, 93], the photosynthesising pelagophyte algae may provide carbon to the host only via their digestion, and therefore may be “farmed” rather than acting as true symbionts. In the laboratory we have observed the pelagophyte algae of *N*. *dutertrei* streaming along the host rhizopodial network, despite a lack of spines for structural support, as is found for the symbionts of spinose species [29, 93, 94]. This would allow optimum algal exposure to sunlight for photosynthesis. Our observations of *N*. *dutertrei* forming a feeding cyst around its shell would pose a complication for these pelagophyte algae. Such a cyst would reduce the light penetration to the algae, even within the rhizopodial network which may not necessarily extend beyond the cyst, and thus also reduce rates of photosynthesis. NanoSIMS analysis of experiments incubating *N*. *dutertrei* with NaH^13^CO_3_ to follow fixed carbon through the putative symbiont-host cells under varying conditions (e.g. [95, 96]) would help to resolve the relationship.

#### Heterotrophic feeding

Previous studies have identified phytoplankton as the main food source for non-spinose planktonic foraminifera such as *N*. *dutertrei* [79, 88] and diatoms in particular have been observed [29, 97]. A diatom food source would, like the pelagophyte algae, be taxonomically assigned to Stramenopile chloroplasts in 16S rRNA gene metabarcoding analysis, and in our dataset ten individual OTUs were assigned to this group. However, 99.9% (DUT55) and 99% (DUT59) of all sequences assigned to the Stramenopile chloroplast group were within a single OTU, and the large numbers of pelagophyte algae, observed in TEM, are highly likely to be the source of this predominant Stamenopile chloroplast OTU. The tiny proportion of sequences assigned to other Stramenopile chloroplasts, and indeed other chloroplast OTUs (< 1%) would suggest that both DUT55 and DUT59 did not contain significant numbers of other Stramenopiles or phytoplankton at the point of collection. We can therefore conclude that they had not recently fed on diatoms, but do not exclude diatoms or other phytoplankton as prey items in this species due to the sporadic nature of feeding, phytoplankton patchiness, seasonality, and the observations of their presence made in previous laboratory studies. Therefore, it remains to be determined whether diatoms and other phytoplankton are significant prey in *N*. *dutertrei* Type Ic.

In one of the two *N*. *dutertrei* individuals (DUT55; Fig 4), 45% of OTUs were of chloroplasts from a plastidic protist, of the phylum Cercozoa, which are the closest relative of foraminifera and radiolarians [98]. Since these organisms are known to glide over surfaces such as sediments and POM rather than be free living in the water column [99], our data suggest that *N*. *dutertrei* does feed within a POM feeding cyst and that Cercozoa are likely to be significant prey of *N*. *dutertrei* Type Ic. The irregular nature of predation would explain the presence of Cercozoa in one individual but not the other, and hence the differences in relative abundance of the putative algal symbiont. Analysis of more individuals is needed for confirmation, but certainly our data suggest that *N*. *dutertrei* Type Ic is omnivorous, and that phytoplankton are not the only component of their diet. A diet including protists has been reported for *Globorotalia menardii* [100]. It has been proposed that protists may be a much more substantial part of the diet in planktonic foraminifera than TEM data suggests due to the rapid digestion of protistan cytoplasm [29]. Whilst our 16S rRNA gene metabarcoding data supports this proposal, a general shortage of available TEM images in the planktonic foraminifera may also be a reason for low numbers of observations of protistan prey.

We propose that *N*. *dutertrei* Type Ic does not feed on bacteria as a primary food source, even though bacterial OTUs account for 2–4% of the total OTUs. Bacteria are ubiquitous in the open ocean, and the numbers of bacteria in POM can be up to 10^8^–10^9^ cells ml^-1^, between 100–10,000-fold higher than in the surrounding water column [53–55]. Therefore, the role of patchiness in the availability of bacterial prey is not as significant as for phytoplankton or protistan prey. If *N*. *dutertrei* Type Ic were preying on bacteria, or phagocytosing POM within the feeding cyst, then far more bacterial OTUs would be expected in the *N*. *dutertrei* profile than were observed in this study.

**Fig 7:**
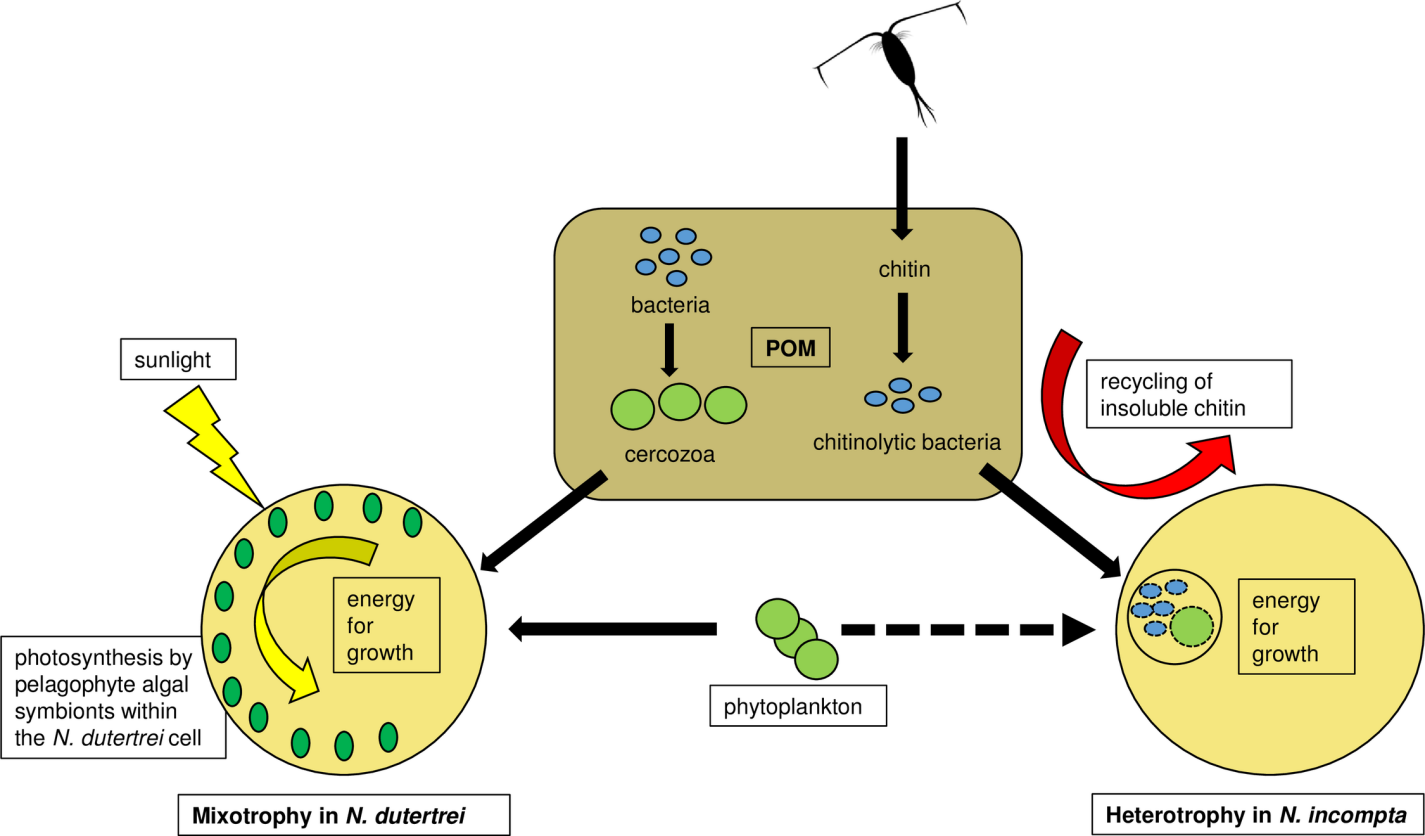
Proposed trophic interactions of *N*. *dutertrei* and *N*. *incompta*. The illustrated trophic interactions are based on 16S rRNA gene metabarcoding performed in this study and laboratory observations reported in the literature. Black arrows indicate direction of heterotrophic carbon flow. We propose that *N*. *dutertrei* Type Ic is a mixotrophic feeder. Pelagophyte algae provide energy for growth through photosynthesis, either via direct consumption of the algae and/or by cross-membrane transport of photosynthates. In addition, *N*. *dutertrei* feeds on other protists and phytoplankton, either from the water column or from inside the POM feeding cyst. For example, within the feeding cyst *N*. *dutertrei* consumes Cercozoa that graze on the POM associated bacteria. In contrast, we propose that *N*. *incompta* Type II is a heterotrophic feeder. As zooplankton moult their chitin carapaces or die, their chitin becomes incorporated into the POM. Here chitinoclastic bacteria (eg. orders Vibrionales and Alteromonadales) break down the chitin to utilise the C and N source. *N*. *incompta* feeds on these orders of bacteria within its POM feeding cyst. *N*. *incompta* also feeds minimally on phytoplankton, but whether from the water column and/or from the feeding cyst is unknown (dashed black arrow).

In our feeding model (Fig 7), we therefore consider *N*. *dutertrei* Type Ic to be a mixotrophic feeder. We hypothesize that it gains energy for growth via both photosynthetic activity of pelagophyte algal symbionts and via heterotrophic feeding on other protists. These include diatoms and plastidic protists such as mixotrophic Cercozoa, which are found either in the water column or within the POM feeding cyst. 18S rRNA gene metabarcoding is needed to elucidate the full profile of protistan prey of this genotype.

#### Trophic interactions of *N*. *incompta*

In contrast to *N*. *dutertei* Type Ic, *N*. *incompta* Type II contains < 0.2% chloroplast OTUs, highlighting the fact that it has evolved an ecological strategy devoid of photosynthetic assistance, and that in the specimens investigated, phytoplankton were not in the recent diet. The OTU profile of *N*. *incompta* Type II is made up almost exclusively of bacterial OTUs, of which 83 to 95% are sequences from the Vibrionales and Alteromonadales orders of class Gammaproteobacteria. A positive amplification bias towards Gammaproteobacteria has been reported in this primer set [67]. However, in a range of surface ocean water samples amplified with these primers, Gammaproteobacteria average around 15% of the assemblage [65], significantly lower than the >83% observed proportion in *N*. *incompta*. Gammaproteobacteria are prevalent in POM [55, 101]. However, the *N*. *incompta* assemblage is significantly different from the POM-associated assemblages investigated from the Bodega Head area [102] where they were collected. It is also significantly different from the POM assemblages further south in the California Current [101], the Santa Barbara Channel [103] and elsewhere [55, 104]. In addition, the *N*. *incompta* bacterial assemblage is also highly divergent from known surface water microbial populations [11, 13, 65, 105, 106]. This high degree of divergence strongly suggests either that the Vibrionales and Alteromonadales are specific prey targeted by *N*. *incompta*, or it simply reflects the nature of the POM available at a specific point in time [107, 108]. For example, Alteromonadales and Vibrionales are significant chitinoclastic orders within the Proteobacteria (they are able to break down chitin, e.g. zooplankton carapaces) [109–111] and hence it is likely that the feeding cyst of each individual foraminifera was made up of chitin-rich POM. This doesn’t exclude the possibility that Alteromonadales and Vibrionales are specific prey, but, if the profile within *N*. *incompta* were a mere reflection of the POM constituents, the 16S rRNA gene profile of *N*. *incompta* should potentially include further chitinoclastic groups. However, it has been demonstrated that Alteromonadales and Vibrionales are prolific producers of substances inhibitory to other bacteria and are the most resilient to such substances [101], suggesting that the presence of these orders within a POM particle may result in a decrease in bacterial diversity. Nevertheless, our data clearly show that *N*. *incompta* does feed on these specific orders of chitinoclastic Gammaproteobacteria, which contribute to the recycling and transfer of C and N from insoluble chitin back into the food web. Recycling of chitin is of huge importance since without it, billions of tonnes of zooplankton carapaces would become buried at the seafloor each year, generating a significant sink for both C and N and changing the dynamics of the global C and N cycles, [112, 113].

There are no reports in the literature pertaining to the preferred diet of *N*. *incompta* specifically. Based on observations of field-collected specimens and laboratory cultures, non-spinose foraminifera are thought to be adapted to a more herbivorous diet than their spinose counter-parts [29, 52]. Throughout the northeast Pacific, *N*. *incompta* is reported to prefer a stratified water column and to sit below the thermocline and chlorophyll maximum (generally below 30 m, averaging between 50 and 100 metres), slightly deeper in the water column than *N*. *dutertrei* [83, 85]. This preferred depth agrees with data from the subtropical northeast Atlantic, where the average depth of *N*. *incompta* was also situated below the chlorophyll maximum, at about 80 m [114]. At such a position in the water column, phytoplankton would still be an available food source, as would bacteria and POM-associated organisms sinking from the productive waters above. It is perhaps surprising therefore that so few chloroplast OTUs (< 0.2%) were observed in *N*. *incompta*. However, our TEM observations indicate that *N*. *incompta* Type II probably does graze on phytoplankton (Fig 5C), albeit to a limited extent, but this may depend on seasonality and phytoplankton availability. Certainly, the preferred depth of *N*. *incompta* during the summer months appears to shoal a little to the base of the chlorophyll maximum, but at lower abundance [85]. In our feeding model, we hypothesize that *N*. *incompta* Type II is a heterotrophic feeder, grazing predominantly on bacteria and to a more limited extent on phytoplankton (Fig 7). The metabarcoding data reveal that *N*. *incompta* can graze principally on chitinoclastic bacteria in the POM, thereby contributing to the recycling of C and N from insoluble chitin. As for *N*. *dutertrei*, 18S rRNA gene metabarcoding is needed to elucidate more fully the eukaryotic prey, if any, of this genotype.

#### Ecological data enhancement of planktonic foraminiferal global distribution and abundance models

Oceanic investigations and both laboratory and modelling studies have demonstrated that in stratified oligotrophic open-ocean waters, mixotrophs [115–117] dominate the protist assemblages. Here they can outcompete purely photo- or heterotrophic organisms, and sustain the functioning of these ecosystems [7, 118–120]. Stratified water masses are currently expanding [121] and are likely to become more common with climate change [122]. Many tropical/subtropical symbiont-bearing foraminifera therefore, would experience an increase in their potential habitat and abundance as these oligotrophic regions expand, and the results of a study using an ecophysiological model, FORAMCLIM, confirm this [31, 32]. This is in stark contrast to a decline in habitat and abundance predicted for the more transitional and higher latitude species modelled; *N*. *dutertrei*, *G*. *bulloides*, *N*. *incompta* and *N*. *pachyderma* [32]. Within the context of FORAMCLIM, all four of these species are considered symbiont-barren, and hence, unlike those species projected to succeed, are not mixotrophic. This indicates that mixotrophy might play an important role in enabling planktonic foraminifera to survive under a changing climate.

Our ecological data now begin to question the scope of the ecophysiological data used for modelling the more transitional species such as *N*. *dutertrei* and *G*. *bulloides*. Given that in the literature *N*. *dutertrei* is referred to as having facultative algal symbionts [29, 49, 51], we now build on that knowledge by demonstrating that *N*. *dutertrei* Type Ic consistently contains large numbers of putative algal symbionts. In addition, we have previously shown that *G*. *bulloides* Type IId, considered symbiont barren, [123, 124] contains large numbers of endobiotic cyanobacteria, whose role is yet to be established [59]. The metabolic contribution to the host foraminifera by these endobiotic algae and cyanobacteria needs to be assessed for all genotypes, and physiological rates of photosynthesis and respiration added to the ecophysiological parameters of the FORAMCLIM model. Adding such data will increase the accuracy of the projections of future distribution and abundance of foraminiferal morphospecies and genotypes, and their likely contribution to calcite export to the deep ocean and the C-cycle.

In contrast, our data demonstrate that the assumed symbiont-barren status of *N*. *incompta* in the FORAMCLIM model is accurate. The model’s projected decrease in abundance in *N*. *incompta* is due primarily to increased temperatures at the higher latitudes, but in the high latitude species, food supply is an additional, secondary factor [32]. This result may reflect a lack of mixotrophy in the species found in these regions, or at least reflect the presumed lack of mixotrophy applied in the model. It is yet to be fully investigated whether the high latitude morphospecies and genotypes, particularly of *N*. *pachyderma* contain symbiotic organisms beyond those ordinarily looked for. It is important therefore, given that mixotrophy appears to bring such advantages, to determine comprehensively the trophic status of each morphospecies and genotype of planktonic foraminifera, if we are to accurately predict their decline or success in their changing environment.

## Conclusions

This is the first report providing evidence for distinctly different microbiota in two species of planktonic foraminifera from the same genus, *Neogloboquadrina*. Both species have the similar feeding strategy of forming a feeding cyst of POM in the water column, and yet their trophic interactions are significantly different. *N*. *dutertrei* Type Ic has evolved a probable symbiotic life-style and we report the first genetic information regarding the coccoid putative algal symbionts which we tentatively assign taxonomically to the Pelagophyceae and not the Chrysophyceae as previously reported [29, 49]. Additional 18S rRNA gene sequencing of these algae is required for full confirmation of this taxonomic assignment. In addition, our data show that *N*. *dutertrei* Type Ic feeds heterotrophically on other protists within the POM, but not on bacteria as a major food source. In contrast, *N*. *incompta* Type II is symbiont barren and predominantly feeds on bacteria within the POM. In the light of the recent finding that *G*. *bulloides* Type IId houses *Synechococcus* endobionts [59], we can now conclude that each planktonic foraminiferal morphospecies has most likely evolved specific interactions with bacteria in the water column. Therefore, each palaeoceanographically important morphospecies requires investigation, particularly when more than one genotype exists, to determine whether the trophic interactions of genotypes differs just as their biogeography does and how this impacts on shell geochemistry [28, 125].

In this study, we have demonstrated that 16S rRNA gene metabarcoding of the intracellular DNA of planktonic foraminifera together with TEM methodologies have the potential to provide new insights into the biological associations and seasonal shifts in feeding preferences of ecologically distinct genotypes of planktonic foraminifera. The primers used in this study have subsequently been improved to avoid known PCR biases [65–67] and hence the new improved primers should be utilised in future studies. With the addition of 18S rRNA gene metabarcoding to target prey or symbiotic heterotrophic protists, next-generation DNA sequencing technologies could transform the usefulness and accuracy of planktonic foraminiferal global distribution and seasonality models, and also increase the accuracy of palaeoproxies by providing essential ecological information currently unavailable [31, 32, 57, 125].

## Supporting information

S1 Fig Alpha-Rarefaction curves for each foraminiferal specimen subjected to 16S rRNA gene metabarcoding. The reduction in the gradient of the curves for each individual specimen with increasing sequencing effort demonstrates that the sequencing depth was sufficient to capture the full bacterial diversity present.
(PDF)

S1 Table The numbers of sequences and OTUs generated in individual specimens. OTU numbers for both closed reference picking and de novo picking are shown after removal of contaminants. Note that total numbers of OTUs are not a sum of OTUs across all specimens, as many OTUs are found in more than one specimen.
(DOCX)
