# Supplementary figures and images for: 16S rRNA gene metabarcoding and TEM reveals different ecological strategies within the genus Neogloboquadrina (planktonic foraminifer)
Source: PLoS One. 2018 Jan 29;13(1):e0191653. doi: 10.1371/journal.pone.0191653 (PMC5788372; doi:10.1371/journal.pone.0191653)

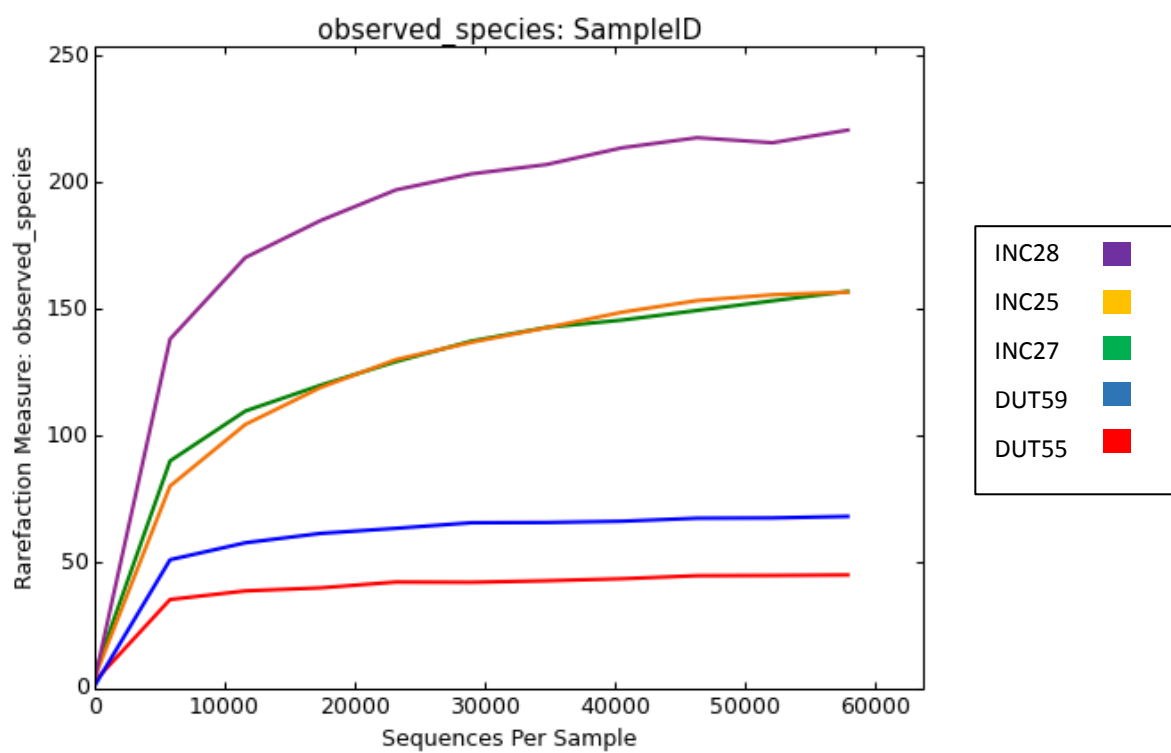

Supplement: S1 Fig — The reduction in the gradient of the curves for each individual specimen with increasing sequencing effort demonstrates that the sequencing depth was sufficient to capture the full bacterial diversity present. (PDF) [file pone.0191653.s001.pdf]
