# Supplementary material for: 16S rRNA gene metabarcoding and TEM reveals different ecological strategies within the genus Neogloboquadrina (planktonic foraminifer)
Source: PLoS One. 2018 Jan 29;13(1):e0191653. doi: 10.1371/journal.pone.0191653 (PMC5788372; doi:10.1371/journal.pone.0191653)
Supplement: S1 Table — OTU numbers for both closed reference picking and de novo picking are shown after removal of contaminants. Note that total numbers of OTUs are not a sum of OTUs across all specimens, as many OTUs are found in more than one specimen. (DOCX) [file pone.0191653.s002.docx]

| Morphospecies | Sample ID | Number of sequences  assigned after closed reference picking | Number of OTUs in closed reference picking | Number of sequences assigned after *de novo* picking | Number of OTUs *de novo* picking |
| --- | --- | --- | --- | --- | --- |
| *N. dutertrei* | DUT55 | 119119 | 49 | 119226 | 76 |
| *N. dutertrei* | DUT59 | 57925 | 68 | 57177 | 63 |
| *N. incompta* | INC25 | 262504 | 183 | 261272 | 224 |
| *N. incompta* | INC27 | 164198 | 190 | 166180 | 173 |
| *N. incompta* | INC28 | 138018 | 238 | 139016 | 265 |
| Total sequences/OTUs |  | 741764 | 346 | 742871 | 403 |
